# Quantitative assessment of invasive mena isoforms (Mena^calc^) as an independent prognostic marker in breast cancer

**DOI:** 10.1186/bcr3318

**Published:** 2012-09-12

**Authors:** Seema Agarwal, Frank B Gertler, Michele Balsamo, John S Condeelis, Robert L Camp, Xiaonan Xue, Juan Lin, Thomas E Rohan, David L Rimm

**Affiliations:** 1 Department of Pathology, Yale University School of Medicine, New Haven, CT 06520, USA; 2 Department of Biology and Koch Institute for Integrative Cancer Research, Massachusetts Institute of Technology, Cambridge, MA 02138, USA; 3 Department of Anatomy and Structural Biology, Gruss Lipper Biophotonics Center, Albert Einstein College of Medicine, Bronx, NY 10461, USA; 4 Department of Epidemiology and Population Health, Albert Einstein College of Medicine, Bronx, NY 10461, USA

## Abstract

**Introduction:**

Mena, an Ena/VASP protein family member, is a key actin regulatory protein. Mena is up-regulated in breast cancers and promotes invasion and motility of tumor cells. Mena has multiple splice variants, including Mena invasive (Mena^INV^) and Mena11a, which are expressed in invasive or non-invasive tumor cells, respectively. We developed a multiplex quantitative immunofluorescence (MQIF) approach to assess the fraction of Mena lacking 11a sequence as a method to infer the presence of invasive tumor cells represented as total Mena minus Mena11a (called Mena^calc^) and determined its association with metastasis in breast cancer.

**Methods:**

The MQIF method was applied to two independent primary breast cancer cohorts (Cohort 1 with 501 and Cohort 2 with 296 patients) using antibodies against Mena and its isoform, Mena11a. Mena^calc ^was determined for each patient and assessed for association with risk of disease-specific death.

**Results:**

Total Mena or Mena11a isoform expression failed to show any statistically significant association with outcome in either cohort. However, assessment of Mena^calc ^showed that relatively high levels of this biomarker is associated with poor outcome in two independent breast cancer cohorts (log rank *P *= 0.0004 for Cohort 1 and 0.0321 for Cohort 2). Multivariate analysis on combined cohorts revealed that high Mena^calc ^is associated with poor outcome, independent of age, node status, receptor status and tumor size.

**Conclusions:**

High Mena^calc ^levels identify a subgroup of breast cancer patients with poor disease-specific survival, suggesting that Mena^calc ^may serve as a biomarker for metastasis.

## Introduction

Many genes implicated in the sequential, multi-step process of metastasis have been identified [1,2]. One of the genes identified is Mena, a member of the Ena/VASP family of proteins, which plays a key regulatory role in actin polymerization [3-6]. It has been shown to be involved in intravasation and motility of tumor cells in model systems [7,8]. In breast cancer tumors, its expression has been shown to be a key element of the tumor microenvironment for metastasis (TMEM), whose density correlates with risk of distant metastasis [9]. Importantly, Mena deficiency in the PyMT mouse breast cancer model suppresses intravasation, eliminates mortality and morbidity, and greatly reduces the frequency of metastatic dissemination to the lung [10].

Mena is alternately spliced to give rise to multiple protein isoforms that are differentially expressed during tumor progression [11,12]. Two of the best characterized isoforms are Mena^INV^, expressed exclusively in invasive tumor cells, and Mena11a, an epithelial-specific isoform expressed in primary breast carcinomas and down-regulated in invasive tumor cells [7]. Mena^INV^, (originally termed Mena^+++^), expression confers a potent pro-metastatic phenotype when expressed in breast cancer cells by potentiating their chemotactic response to epidermal growth factor (EGF), thereby enhancing their ability to engage in efficient streaming motility via increasing their paracrine signaling with macrophages [3,13,14]. The Mena11a, a non-metastatic isoform, contains an alternately-included exon encoding a 21 amino acid insertion located in the carboxy-terminal [7]. Consistent with its down-regulation during tumor progression *in vivo *[11,15], Mena11a is expressed in epithelial-like but not mesenchymal-like tumor cell lines [16], and is down-regulated when human mammary epithelial cells undergo epithelial to mesenchymal transition (EMT) [12]. Mena11a expression in breast cancer cells causes formation of poorly metastatic tumors with a highly epithelial architecture that are not capable of responding to EGF chemotactic cues *in vivo *[14]. Therefore, Mena11a expression positively correlates with, and enforces epithelial non-metastatic phenotypes, and negatively correlates with, and suppresses mesenchymal metastatic phenotypes *in vitro *and *in vivo*.

The mechanistic role of Mena^INV ^raises the hypothesis that measurement of this isoform in tumor tissue could be valuable for prediction of the risk of metastasis. Thus, it is reasonable that the fraction of Mena containing the 11a exon may reflect the abundance of poorly-metastatic tumor cells and, therefore, correlate with decreased metastatic risk. Thus far, no evidence exists indicating that both the INV and 11a exons are included in the Mena mRNA at the same time or expressed at high levels within the same cell. Therefore, the overall fraction of Mena lacking 11a may reflect the presence of cells with the potential to express pro-metastatic Mena isoforms. We describe here a multiplexed quantitative immufluorescence-based method (MQIF) in which the fraction of Mena protein that may promote invasion inferred by subtraction of the non-invasive isoform from the total Mena present in tumors. We call this biomarker Mena^calc ^and in the study reported here relate it to metastasis using risk of death from breast cancer.

## Materials and methods

### Cohorts

This study was conducted using data from two cohorts of breast cancer patients. The first cohort consists of 501 patients who underwent surgery at Yale University Cancer Center/Yale New Haven hospital between1962 and 1982 and had formalin-fixed, paraffin-embedded (FFPE) primary invasive breast tumors available for study. Cohort 2 consists of 296 patients who had surgery for breast cancer at Yale University Cancer Center/Yale New Haven hospital between1976 and 2005 and for whom FFPE tissue was available. Tissue microarrays were constructed in two-fold redundancy for each cohort. Both cohorts have been described previously [17,18]. In both, follow-up information on cases was obtained from the Yale New Haven Tumor Registry, the Yale-New Haven Hospital medical records and the Connecticut Death Records. Tissues were collected in accordance with consent guidelines using protocol number 9500008219 issued to DLR from the Yale Institutional Review Board, most recently reapproved in May 2012.

### Antibodies and multiplexed immunofluorescence staining

The arrays were deparaffinized first by melting at 60°C in an oven equipped with a fan for 20 minutes followed by 2x xylene treatment for 20 minutes each. Slides were then rehydrated and antigen retrieval was done in citrate buffer (pH 6.0) at 97°C for 20 minutes in a PT module (Labvision, Kalamazoo, MI, USA). Endogenous peroxidase was blocked by using 0.3**% **hydrogen peroxide in methanol for 30 minutes in the dark followed by incubation of slides in a blocking buffer (0.3% bovine serum albumin in TBST (0.1 mol/L of TRIS-buffered saline (pH 7.0) containing 0.05% Tween-20)) for 30 minutes at room temperature. Slides were incubated with a cocktail of mouse anti-pan-Mena (1:1,000 dilution; BD Biosciences, San Jose, CA, USA, catalog number 610693) mixed with rabbit anti-Mena11a (1:500 dilution of 1 mg/ml stock; generated in the laboratory of FBG) in the blocking buffer overnight at 4°C. After washing away the primary antibodies, slides were incubated with secondary antibody (goat anti-rabbit conjugated to horseradish peroxidase, Jackson ImmunoResearch Laboratories Inc., West Grove, PA, USA) to target Mena11a for one hour. After washing, slides were incubated with biotinylated tyramide (Perkin Elmer, Waltham, MA, USA) diluted as 1:50 in amplification buffer (Perkin Elmer) for 10 minutes. After washing, peroxidase activity was quenched by 2x treatment with benzoic hydrazide (100 mM in PBS; Sigma-Aldrich, St. Louis, MO, USA) with 50 mM hydrogen peroxide for seven minutes each. After washing, slides were incubated for an hour with goat anti-mouse envision (DAKO, Carpinteria, CA, USA) followed by treatment with a chicken anti-Pan cytokeratin (1:100, generated in house) for 2 h at room temperature. Slides were washed and then incubated with goat anti-chicken conjugated to Alexa546 (Invitrogen, Grand Island, NY, USA) to visualize cytokeratin ) and streptavidin conjugated to CY7 (750 nm, Invitrogen, Grand Island, NY, USA) to visualize Mena11a for an hour. After washing, slides were treated with CY5 conjugated tyramide (1:50 dilution; Perkin Elmer, Waltham, MA, USA) in amplification buffer for 10 minutes to visualize pan-Mena. Slides were mounted with ProLong gold mixed with DAPI (Molecular Probes, Grand Island, NY, USA). Serial sections of the index array used for assay standardization [18] were stained alongside each cohort to assess the assay reproducibility. An additional serial section of the index array was stained with each experiment with no primary antibodies as a negative control.

### Automated quantitative analysis (AQUA) and calculation of Mena^calc ^fraction

The AQUA technology (HistoRx, Branford, CT, USA) allows quantitative measurement of biomolecules in sub-cellular compartments as described previously [19,20]. Briefly, a series of monochromatic images for each histospot was captured using a PM-2000 microscope (HistoRx, Branford, CT, USA) equipped with an automated stage. A binary 'tumor mask' was created using cytokeratin staining of the histospot representing only epithelial cells and excluding stromal features. AQUA scores for both pan-Mena and Mena11a were calculated by dividing the signal intensity by the area of the specific compartment (in this case within the tumor mask area). Normalized AQUA scores for both targets (pan-Mena and Mena11a) were used to calculate the Mena^calc ^fraction for each histospot by subtracting the z score of Mena11a from the z score of pan-Mena as described below.

### Statistical analysis

Pearson's correlation coefficient (*R*) was used to assess the reproducibility of the multiplexed assay between near-serial sections of the index array. Based on extensive experience in our laboratory, an *R^2 ^*value greater than 0.4 was considered acceptable for both inter- and intra-array reproducibility. For both cohorts, pan-Mena, Mena11a AQUA scores, and Mena^calc ^values from two independent cores for each histospot were averaged and the averages were used for final analysis. Both cohorts were independently divided into quartiles based on the distribution of Mena^calc ^values in the respective cohorts. Within each cohort, the survival rates in the lowest three quartiles were overlapping so these three quartiles combined into a single group (Q1 to 3, data not shown). The top quartile (Q4) was compared with the lowest three quartiles for disease-specific survival differences using the log-rank test. A multivariate Cox proportional hazards analysis was used to examine the prognostic value of Mena^calc ^while controlling for other common prognostic factors as well as potential confounders. Since similar differences between Mena^calc ^groups were observed in both cohorts, we combined the two cohorts for the multivariate analysis to gain efficiency while stratifying on the cohort to allow the two cohorts to have different baseline survival functions, as the Kaplan-Meier survival curves suggested that Cohort 2 had better survival. All *P-*values were two-sided, and *P*-values <0.05 were considered to be statistically significant. All statistical analyses were done using SAS 9.2 (SAS Institute, Cary, NC, USA).

## Results

### Development of multiplexed assay for Mena^calc ^

**Figure 1:**
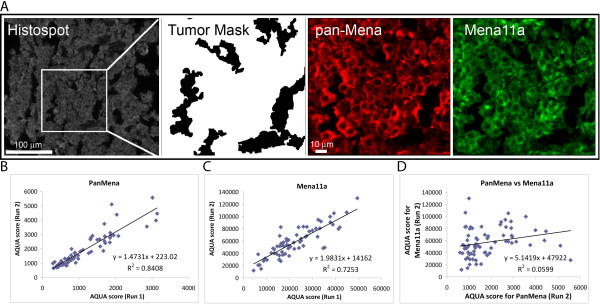
**Development of a robust and reproducible MQIF assay to assess Mena^calc^**. **A**. Representative tumor histospot with Pancytokeratin staining used to define the tumor mask, pan-Mena (red) and Mena11a (green) showing a cytoplasmic staining pattern. Scale bar is shown at the left bottom of the panels. **B**, **C**. Reproducibility of MQIF Mena^calc ^assay. Linear regression between two independent runs of pan-Mena (B) and Mena11a (C) multiplexed on serial sections of an index TMA have a high correlation (*R*^2 ^= 0.84 and 0.73 respectively). **D**. Linear regression between pan-Mena and Mena11a on an index TMA slide shows no correlation (*R*^2 ^= 0.06).

We developed and validated a method of MQIF to assess the relative abundance of total Mena and the anti-metastatic Mena11a by multiplexing pan-Mena antibody with antibody against Mena11a, and then subtracting the Mena11a from the total Mena as measured by a "pan-Mena" antibody (that recognizes all known Mena protein isoforms). Pan-cytokeratin staining was used to define the epithelial component of the tumor area in which the signal intensity of total Mena and Mena11a was assessed. As expected, a cytoplasmic localization pattern was observed for both pan-Mena and Mena11a (Figure 1A). Inter-array reproducibility of the MQIF assay was evaluated by staining serial sections of an index array TMA (tissue microarray) consisting of a small subgroup of breast cancer patients used as a control array for each experiment. This yielded Pearson's *R^2 ^*values of 0.841 for pan-Mena and 0.725 for Mena11a, indicative of good reproducibility (Figure 1B, C). Further, cross-reactivity of the two antibodies was determined using linear regression between the AQUA scores of both antibodies for the same index slide. As shown in Figure 1D, there was no correlation (*R^2 ^*= 0.059) between pan-Mena and Mena11a, suggesting assay isoform specificity that total Mena expression levels are independent of the frequency of 11a exon inclusion.

### Reproducibility and heterogeneity of Mena^calc ^expression in the two independent cohorts

**Figure 2:**
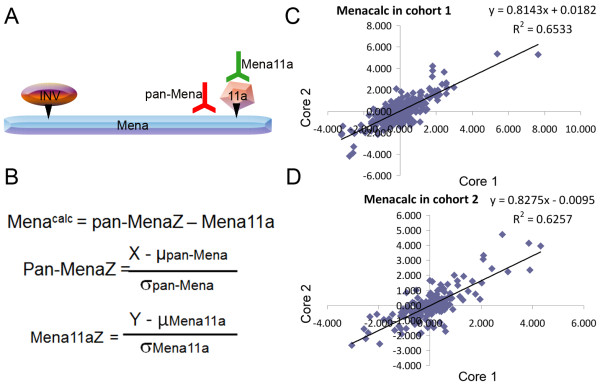
**Generation of Mena^calc ^scores and assessment of tumor heterogeneity in two independent breast cancer cohorts**. **A**. Schematic representation of Mena, alternatively spliced inclusion of exons for Mena isoforms and binding sites for pan-Mena and Mena11a antibodies. **B**. Equation for the calculation of Mena^calc ^scores and equations used to calculate Z scores for pan-Mena and Mena11a. × is the AQUA score for pan-Mena, Y is the AQUA score for Mena11a, μ is the mean of the AQUA scores for pan-Mena or Mena11a in each independent cohort and σ is standard deviation for pan-Mena or Mena11a AQUA scores for each independent cohort. **C, D**. Tumor heterogeneity of Mena^calc ^in two independent cores of 501 individual patients from Cohort 1 (C) and 296 patients from Cohort 2 (D).

The different isoforms of Mena are shown schematically in Figure 2A. The relative amount of Mena lacking 11a was calculated by first converting the AQUA scores of both pan-Mena and Mena11a to Z scores to normalize the AQUA scores to the same scale. Then Mena^calc^, a measure of the abundance of Mena lacking its anti-metastatic Mena11a isoform, was calculated by simple subtraction of Z scores of Mena11a from the total Mena (Figure 2B). The multiplexed assay was applied to the two primary breast cancer cohorts on TMAs. Tumor heterogeneity was evaluated by comparing Mena^calc ^values in two different cores (histospots) from the same tumor blocks. This showed significant correlation between histospots (*R^2 ^*= 0.653 for cohort 1 and 0.626 for cohort 2), as shown in Figure 2C, D. Based on our experience with TMA and AQUA technology, two independent 0.6 mm cores from each patient has been considered representative of a whole tissue section. While this is clearly a function of the heterogeneity of the marker considered and the tumor type, the use of two cores is often the standard for TMA based studies [21,22].

### Clinicopathological characteristics

Clinicopathological characteristics of both cohorts and of the combination of the two are presented in Table 1. Compared to cohort 1, the tumors in cohort 2 were smaller in size and more likely to be ER^+^, PR^- ^and Her2^-^.

**Table 1:** Clinicopathological characteristics of Yale cohorts

	Cohort 1	Cohort 2	Combined
**Characteristic **	**N (%)**	**N (%)**	**N (%)**
All patients	501	296	797
Age (y)			
<50	146 (29.1)	96 (32.4)	242 (30.4)
≥50	355 (70.8)	200 (67.6)	555 (69.6)
Nodal Status			
positive	267 (53.2)	59 (19.9)	326 (40.9)
negative	234 (46.7)	160 (54.1)	394 (49.4)
unknown	0 (0)	77 (26.0)	77 (9.7)
Tumor Size (mm)			
≤2	155 (30.9)	148 (50.0)	303 (38.0)
2 to 5	251 (50.1)	78 (26.4)	388 (48.7)
≥5	55 (11.0)	4 (1.3)	
unknown	40 (8.0)	66 (22.3)	106 (13.3)
ER (IHC)			
positive (1 to 3)	251 (50.1)	164 (55.4)	415 (52.1)
negative (0)	234 (46.7)	97 (32.8)	331 (41.5)
unknown	16 (3.2)	35 (11.8)	51 (6.4)
PR (IHC)			
positive (1 to 3)	241 (48.1)	33 (11.1)	274 (34.4)
negative (0)	233 (46.5)	221 (74.7)	454 (57.0)
unknown	27 (5.4)	42 (14.2)	69 (8.6)
HER2 (IHC)			
positive (2 to 3)	86 (17.2)	30 (10.1)	116 (14.6)
negative (0 to 1)	393 (78.4)	213 (72.0)	606 (76.0)
unknown	22 (4.4)	53 (17.9)	75 (9.4)
Follow-up (m)			
median (range)	105.1 (2.39 to 498.03)	120.0 (3 to 385)	113.0 (2.4 to 250)

IHC, immunohistochemistry; m, months; N, number; y, years

### Association of Mena^calc ^with disease-specific survival

**Figure 3:**
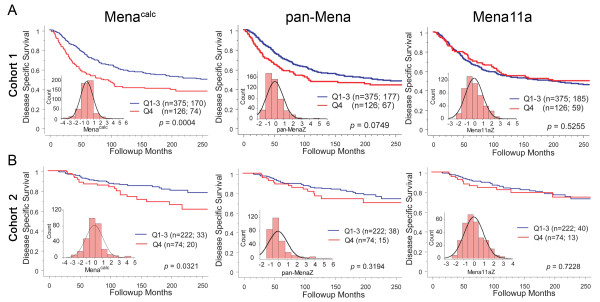
**Kaplan-Meier analysis for Mena^calc^, pan-Mena and Mena11a in two primary breast cancer cohorts**. Each cohort was divided into quartiles and disease-specific survival of the highest quartile of expression was compared to the remaining patient population. Inset is the histogram distribution of Mena^calc ^or Z scores for each antibody. The number of patients in each group is shown followed by the number of patient deaths due to disease. Log-rank *P-*values are shown for 20-year follow-up. Only Mena^calc ^scores are statistically significant in each cohort (*P *= 0.0004 and 0.0321, respectively).

**Figure 4:**
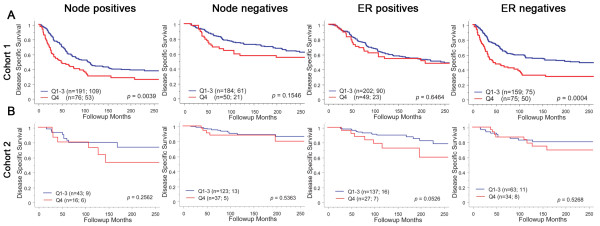
**Kaplan-Meier analysis for Mena^calc ^in sub-populations based on nodal status and ER status in two primary breast cancer cohorts**. Each cohort was divided into quartiles and disease specific survival of the highest quartile of expression was compared to the remaining patient population. The number of patients in each group is shown followed by the number of patient deaths due to disease. Log-rank *P-*values are shown for 20-year follow-up. Only Mena^calc ^scores in node positive and ER negative sub-populations are statistically significant in cohort 1 (*P *= 0.0039 and 0.0004 respectively).

Mena^calc ^was analyzed using averaged AQUA scores of two independent cores for both cohorts. We evaluated the prognostic impact of Mena^calc ^in Cohort1 using 20 years of follow-up for survival of patients by dividing the population (501 patients) into quartiles. The highest quartile (Q4) of Mena^calc ^was strongly associated with poor outcome (log rank *P *= 0.0004, Figure 3A). The lowest three quartiles (Q1 to 3) did not differ between each other in their association with survival (data not shown) and, therefore, we combined them for further analysis. Pan-Mena and Mena11a quartiles alone were not associated with survival (Figure 3A) (log rank *P *= 0.0749 and *P *= 0.5255, respectively). Cohort 2, consisting of 296 patients, yielded similar results. Again, the highest quartile of Mena^calc^, but not pan-Mena or Mena11a, was able to stratify patients for poor outcome (log rank *P *= 0.0321, Figure 3B). Cohort 2 consists of patients from 1976 to 2005 and thus shows fewer events (53 for Cohort 2 vs 244 for Cohort 1) as shown in Figure 3A, B and thus is less well-powered. Additionally, we assessed the impact of Mena^calc ^on survival within subgroups of both cohorts. Mena^calc ^was prognostic in the node positive subset with a log rank *P-*value of 0.0039, but not in node negative patients (Figure 4A). Likewise, Mena^calc ^showed prognostic value among the ER negative patient group (log rank *P *= 0.0004), but not in ER positive patients (log rank *P *= 0.5651). We evaluated the same subgroups in Cohort 2 but failed to see any significant differences within these subgroups (Figure 4B).

### Multivariate analysis of the combined cohort

The cohorts were combined for multivariate analysis, in which a multivariate Cox proportional hazards analysis was used to examine the prognostic value of Mena^calc ^while controlling for other common prognostic factors as well as potential confounders. Mena^calc ^retained its prognostic value such that patients in the highest quartile had a 60% increase in risk of breast cancer death compared to those in the lowest three quartiles (hazard ratio, HR = 1.597; 95% CI = 1.20 to 2.13; *P *= 0.0015, Table 2). The linear trend in risk across Mena^calc ^was statistically significant: HR = 1.211 (1.08 to 1.36), *P *= 0.00164. In addition to Mena^calc^, age, nodal status, nuclear grade and tumor size were significantly associated with risk of death from breast cancer (Table 2 and Additional file 1, Table S1).

**Table 2:** Risk of death from breast cancer in association with Mena^calc^

Combined Cohort 1 and 2 (n = 608; 231)*			
**Variable**		**HR (95% CI)**	***P*-value**
Age			
	<50	1.00	0.0738
	>50	1.316 (0.97 to 1.78)	
Tumor size			
	<2 cm	1.00	**<0.0001**
	2 to 5 cm	2.127 (1.57 to 2.88)	
Nuclear grade			
	low	1.00	
	high	1.250 (0.93 to 1.68)	**0.0397**
	ND	2.414 (1.10 to 5.32)	
Nodal status			
	Positive	2.161 (1.60 to 2.92)	**<0.0001**
	Negative	1.00	
ER			
	Positive	0.755 (0.57 to 1.00)	0.0549
	Negative	1.00	
PR			
	Positive	0.899 (0.68 to 1.19)	0.4602
	Negative	1.00	
Her2			
	Positive	1.360 (0.96 to 1.93)	0.0834
	Negative	1.00	
Mena^calc^			
	1 to 3 Q	1.00	
	4 Q	1.597 (1.20 to 2.13)	**0.0015**
Mena^calc^	continuous	1.211 (1.08 to 1.36)	**0.0016**

The *P-*values are shown for a 20-year follow-up and bold numbers indicate statistically significant *P*-value.

*The number of patients in each cohort is shown followed by the number of patient deaths due to disease.

## Discussion

From a mechanistic standpoint, measurement of the relative abundance of Mena isoforms associated specifically with poor or high metastatic potential represents an attractive approach for prediction of metastasis. In this paper, we describe the development of a marker generated using a subtractive approach to estimate the abundance of Mena lacking its anti-Metastatic Mena11a isoform. The advantage of this approach is the ability to infer the actual (Mena11a) or potential (Mena^calc^) abundance of anti- or pro-metastatic Mena isoforms in a single assay.

Another related mechanism-based assay used to assess metastasis risk is measurement of TMEM density [9]. TMEM counts performed in a small case control study of 30 pairs of invasive breast cancer patients in which half of them (30) developed distant metastasis, while another 30 patients did not. TMEM counts were significantly higher in the patient group that developed distant metastasis compared to the patient group that had only localized breast tumor (*P *= 0.00006), independent of other clinicopathologic variables [9]. Roughly, the risk of distant metastases doubled as for every 10 TMEMs measured in the area of patient tissue analyzed. Expression of Mena^INV^, but not Mena 11a, correlates with TMEM density supporting the hypothesis that Mena11a is not involved in the assembly of TMEM intravasation sites [15]. While it will be interesting to compare the TMEM assay with the Mena^calc ^method, such a comparison is technically challenging. The TMEM assay requires larger tissue specimens (whole slides) to count the morphologically defined triad of a Mena-expressing tumor cell, macrophage and blood vessel all contacting each other. While the Mena^calc ^method may be assessed on whole slides, this initial effort is limited to TMA-based material. While we believe that our MQIF method will be suitable for clinical usage to identify patients that are at high risk for developing metastasis, it awaits testing on routine whole slide specimens. However, our results on TMAs suggest that this approach may permit higher throughput, less sensitivity to heterogeneity and, perhaps, increased sensitivity given the larger tissue requirements and more laborious method required for TMEM counts.

Previously, we reported a significant correlation between Mena^INV ^mRNA levels measured in fine needle aspirate biopsies of clinical samples and TMEM frequency in histological sections from the same patient [15]. Clearly it will be interesting to evaluate how Mena^calc ^compares to Mena^INV ^levels; however, such analyses will require development of a Mena^INV^-specific antibody that can be used for tissue staining. We predict, however, that the two metrics (Mena^calc ^and Mena^INV^) will likely convey different information. Mena^calc ^measures the total amount of all Mena isoforms expressed that do not contain the 11a exon (pan-Mena-Mena11a). Therefore, Mena^calc ^represents all Mena isoforms other than the anti-invasive/metastatic Mena11a. While Mena^INV ^is expressed during tumor progression and has pro-metastatic activity, other Mena isoforms lacking the INV exon, including Mena "classic", which lacks all known alternately-included exons, Mena++ and Mena+ [3], are still expressed in tumors and in invasive tumor cells isolated from mouse breast cancer models [11]. Therefore, we do not propose that Mena^calc ^is a surrogate measure for Mena^INV^. Instead, we propose that this metric measures the loss of the anti-metastatic activity of Mena11a. Whether a combined evaluation of Mena^calc ^and Mena^INV ^provides more powerful prognostic information than either alone will be an important focus of future efforts.

While the results of this investigation suggest that our assay predicts tumor aggressiveness, this study has a number of limitations. First, we used disease-specific survival as a surrogate marker for metastasis. We believe this is reasonable since essentially every death from breast cancer is due to metastasis. Another, more significant weakness of the study is that the cohorts are both retrospective collections from a single institution. Furthermore, this is a *post-hoc *study design and the conclusions will need to be validated on other cohorts prior to clinical application. The fact that Mena^calc ^is able to stratify patients in the multivariate Cox model analysis on the combined cohorts provided increases our confidence in the assay. However, it will be important to confirm this observation in larger, prospectively collected, multi-institutional cohorts.

## Conclusions

In conclusion, we have developed a robust and reproducible QMIF assay for assessing the abundance of Mena lacking the anti-metastatic 11a isoform represented as Mena^calc^. Our results indicate that Mena^calc ^can be used successfully to stratify patients into high- and low-risk for developing metastasis. This test may have value in node positive, ER negative patients which are not addressed by current, commonly used prognostic assays. Once validated in larger multi-institutional cohorts, this assay may provide additional information to assist breast cancer oncologists in selecting appropriate therapy for their patients.

## Abbreviations

AQUA: Automated quantitative analysis; CI: confidence interval; EGF: epidermal growth factor; EMT: epithelial to mesenchymal transition; ER: estrogen receptor; FFPE: formalin-fixed paraffin embedded; HR: hazard ratio; MQIF: Multiplexed quantitative immunofluorescence; PR: Progesterone receptor; Q: quartile; TMA: tissue microarray; TMEM: Tumor microenvironment for metastasis

## Competing interests

Drs. Condeelis and Gertler are stockholders in Metastat, a company with the exclusive license to the Mena patent suite. Drs. Rohan and Rimm serve as consultants to Metastat. Dr. Rohan is a stockholder of Metastat. Drs. Rimm and Camp are founders, consultant and stockholders of HistoRx.

## Authors' contributions

SA, DLR, FBG and JSC participated in the concept, design and coordination of the study, and the drafting of the manuscript. SA was also involved in the execution, acquisition, analysis and interpretation of all data. DLR participated in providing financial support, patient cohorts, data analysis and interpretation of all data. FBG and JSC provided the Mena 11a antibody used in this work. TER, RLC, XX and JL helped with all statistical analysis and the drafting of the manuscript. MB helped in generating and validating the Mena 11a antibody and helped in drafting the manuscript. All authors read and approved the final manuscript for publication.

## Supplementary Material

Additional file 1 **Table S1: Subgroup analysis of combined cohort for the risk of death from breast cancer in association with Mena^calc^**. The *P-*values are shown for a 20-year follow-up. Table showing correlation of Mena^calc ^with risk of death from breast cancer among node negative, node positive, ER negative and ER positive subgroups in a multivariate Cox proportional hazards analysis. Click here for file
