# Supplementary material for: Quantitative assessment of invasive mena isoforms (Menacalc) as an independent prognostic marker in breast cancer
Source: Breast Cancer Res. 2012 Sep 12;14(5):R124. doi: 10.1186/bcr3318 (PMC3962029; doi:10.1186/bcr3318)
Supplement: Additional file 1 — Table S1: Subgroup analysis of combined cohort for the risk of death from breast cancer in association with Menacalc. The P-values are shown for a 20-year follow-up. Table showing correlation of Menacalc with risk of death from breast cancer among node negative, node positive, ER negative and ER positive subgroups in a multivariate Cox proportional hazards analysis. [file bcr3318-S1.DOCX]

Additional file 1: Table S1

Subgroup analysis of combined cohort for the risk of death from breast cancer in association with Mena^calc^. The p values are shown for 20 year follow-up.

|  | | **Node negative (n = 331; 79)*** | | **Node positive (n=277; 152)*** | | **ER negative (n = 306; 114)*** | | **ER positive (n = 302; 117)*** | |
| --- | --- | --- | --- | --- | --- | --- | --- | --- | --- |
| **Variable** | | **HR (95% CI)** | **p-Value** | **HR (95% CI)** | **p-Value** | **HR (95% CI)** | **p-Value** | **HR (95% CI)** | **p-Value** |
| Age |  |  |  |  |  |  |  |  |  |
|  | <50 | 1.00 |  | 1.00 |  | 1.00 |  | 1.00 |  |
|  | >50 | 1.346 (0.81-2.23) | 0.2488 | 1.292 (0.88-1.89) | 0.1882 | 1.426 (-.97-2.10) | 0.0678 | 1.029 (0.64-1.67) | **0.9064** |
| Tumor Size | |  |  |  |  |  |  |  |  |
|  | <2 cm | 1.00 |  | 1.00 |  | 1.00 |  | 1.00 |  |
|  | > 2 cm | 2.458 (1.51-4.01) | **0.0003** | 1.924 (1.31-2.82) | **0.0008** | 2.082 (1.37-3.16) | **0.0006** | 2.067 (1.33-3.22) | **0.0013** |
| Nuclear Grade | |  |  |  |  |  |  |  |  |
|  | low | 1.00 |  | 1.00 |  | 1.00 |  | 1.00 |  |
|  | high | 0.893 *0.47-1.71) | 0.1403 | 1.413 (1.00-1.99) | ND | 1.087 (0.73-1.62) | 0.7433 | 1.554 (0.99-2.44) | 0.0313 |
|  | ND | 2.183 (0.97-4.91) |  | Not Estimable |  | 2.02 (0.26-15.53) |  | 2.431 (1.02-5.81) |  |
| Nodal Status | | NA |  | NA | NA |  |  |  |  |
| Positive | |  |  |  |  | 2.343 (1.49-3.68) | **0.0002** | 2.074 (1.35-3.18) | **0.0009** |
| Negative | |  |  |  |  | 1.00 |  | 1.00 |  |
| ER |  |  |  |  |  | NA | NA | NA | NA |
| Positive | | 0.832 (0.48-1.43) | 0.5045 | 0.701 (0.49-1.00) | **0.0485** |  |  |  |  |
| Negative | | 1.00 |  | 1.00 |  |  |  |  |  |
| PR |  |  |  |  |  |  |  |  |  |
| Positive | | 0.870 (0.51-1.48) | 0.6089 | 0.883 (0.63-1.24) | 0.4754 | 0.715 (0.46-1.10) | 0.1288 | 1.065 (0.71-1.60) | 0.7616 |
| Negative | | 1.00 |  | 1.00 |  | 1.00 |  | 1.00 |  |
| Her2 |  |  |  |  |  |  |  |  |  |
| Positive | | 0.893 (0.47-1.71) | 0.3209 | 1.329 (0.84-2.09) | 0.2196 | 1.255 (0.81-1.94) | 0.3094 | 1.307 (0.73-2.34) | 0.3682 |
| Negative | | 1.00 |  | 1.00 |  | 1.00 |  | 1.00 |  |
| Mena^calc^ |  |  |  |  |  |  |  |  |  |
|  | 1-3 Q | 1.00 |  | 1.00 |  | 1.00 |  | 1.00 |  |
|  | 4 Q | 1.843 (1.11-3.07) | **0.0186** | 1.456 (1.02-2.08) | **0.0394** | 1.546 (1.06-2.27) | **0.0256** | 1.494 (0.94-2.38) | 0.0898 |

*The number of patients in each cohort is shown followed by the number of patient deaths due to disease.
